# Relationship of Predicted Risk of Developing Invasive Breast Cancer, as Assessed with Three Models, and Breast Cancer Mortality among Breast Cancer Patients

**DOI:** 10.1371/journal.pone.0160966

**Published:** 2016-08-25

**Authors:** Mark E. Sherman, Laura Ichikawa, Ruth M. Pfeiffer, Diana L. Miglioretti, Karla Kerlikowske, Jeffery Tice, Pamela M. Vacek, Gretchen L. Gierach

**Affiliations:** 1 National Cancer Institute, Division of Cancer Prevention and Division of Cancer Epidemiology and Genetics, Bethesda, Maryland, United States of America; 2 Group Health Research Institute, Group Health Cooperative, Seattle, Washington, United States of America; 3 Division of Cancer Epidemiology and Genetics, Bethesda, United States of America; 4 Group Health Research Institute, Group Health Cooperative, Seattle, Washington, United States of America; 5 University of California Davis, Department of Public Health, Davis, California, United States of America; 6 University of California San Francisco, Department of Medicine and Department of Epidemiology/Biostatistics, San Francisco, California, United States of America; 7 University of California at San Francisco, Department of Medicine, San Francisco, United States of America; 8 University of Vermont School of Medicine, Department of Medical Biostatistics, Burlington, Vermont, United States of America; Centro per lo Studio e la Prevenzione Oncologica, ITALY

## Abstract

**Purpose:**

Breast cancer risk prediction models are used to plan clinical trials and counsel women; however, relationships of predicted risks of breast cancer incidence and prognosis after breast cancer diagnosis are unknown.

**Methods:**

Using largely pre-diagnostic information from the Breast Cancer Surveillance Consortium (BCSC) for 37,939 invasive breast cancers (1996–2007), we estimated 5-year breast cancer risk (<1%; 1–1.66%; ≥1.67%) with three models: BCSC 1-year risk model (BCSC-1; adapted to 5-year predictions); Breast Cancer Risk Assessment Tool (BCRAT); and BCSC 5-year risk model (BCSC-5). Breast cancer-specific mortality post-diagnosis (range: 1–13 years; median: 5.4–5.6 years) was related to predicted risk of developing breast cancer using unadjusted Cox proportional hazards models, and in age-stratified (35–44; 45–54; 55–69; 70–89 years) models adjusted for continuous age, BCSC registry, calendar period, income, mode of presentation, stage and treatment. Mean age at diagnosis was 60 years.

**Results:**

Of 6,021 deaths, 2,993 (49.7%) were ascribed to breast cancer. In unadjusted case-only analyses, predicted breast cancer risk ≥1.67% versus <1.0% was associated with lower risk of breast cancer death; BCSC-1: hazard ratio (HR) = 0.82 (95% CI = 0.75–0.90); BCRAT: HR = 0.72 (95% CI = 0.65–0.81) and BCSC-5: HR = 0.84 (95% CI = 0.75–0.94). Age-stratified, adjusted models showed similar, although mostly non-significant HRs. Among women ages 55–69 years, HRs approximated 1.0. Generally, higher predicted risk was inversely related to percentages of cancers with unfavorable prognostic characteristics, especially among women 35–44 years.

**Conclusions:**

Among cases assessed with three models, higher predicted risk of developing breast cancer was not associated with greater risk of breast cancer death; thus, these models would have limited utility in planning studies to evaluate breast cancer mortality reduction strategies. Further, when offering women counseling, it may be useful to note that high predicted risk of developing breast cancer does not imply that if cancer develops it will behave aggressively.

## Introduction

Improving methods to predict risk of developing breast cancer and communicating this information to women would enable them to make more informed screening and prevention choices.[1–3] Models that predict breast cancer risk incorporate multiple factors, including age, family history of breast cancer and menstrual, reproductive and medical history.[4–14] Generally, these models accurately predict the number of breast cancers that will develop within groups of women in specified populations, and therefore are useful for estimating sample sizes required in prevention trials.[4, 7] However, the ability of the risk models to discriminate between women who will and will not develop breast cancer is limited; approximately 60% of women that develop cancer have higher risk scores than women who remain cancer free.[4, 7]

Breast cancer risk factor associations vary by tumor subtypes.[13, 15–17] Given that risk prediction models were developed to estimate overall invasive breast cancer risk in populations screened with mammography, these models may demonstrate greater accuracy in predicting risk of estrogen receptor (ER) positive cancers and indolent tumors, which are frequent in this setting.[18–22] However, the performance of these models in predicting risk of fatal breast cancers, as would be needed to design trials to reduce breast cancer mortality, is largely unknown. Given that only a limited number of analyses have examined associations of predicted risk of developing breast cancer with breast cancer mortality,[23, 24] we evaluated these relationships among breast cancer cases in a large U.S. registry of breast imaging facilities for three breast cancer risk models used in the U.S.: Breast Cancer Surveillance Consortium (BCSC) 1-year risk model developed by Barlow et al[8] (referred to as BCSC-1), BCRAT developed by Gail[9, 10, 14] and the BCSC 5-year risk model proposed by Tice et al[5] (referred to as BCSC-5). Although these models were developed to predict overall breast cancer incidence, i.e. risk of developing any breast cancer, rather than tumor subtype or behavior, understanding how these models perform with respect to these factors is important for planning prevention research and counseling patients. Further, assessing the performance of existing models to predict risk of fatal breast cancers among cases, and exploring reasons for the observed performance may help identify future needs and directions for breast cancer risk modeling.

### Participants and Methods

#### Participants

This analysis includes data collected by the BCSC in seven population-based U.S. mammography registries as compiled and analyzed at the Statistical Coordinating Center (SCC).[25] The BCSC registries are linked to the Surveillance, Epidemiology, and End Results (SEER) program, state cancer registries and pathology databases to identify cancer diagnoses.

We identified 43,489 women aged 35 to 89 years diagnosed with a first primary invasive breast cancer from 1996 to 2007 for whom risk factor information (mainly pre-diagnostic) was available (see below). Women with a history of ductal carcinoma *in-situ* or use of chemoprevention were excluded. Of the 43,489 women, we excluded 3,484 with a self-reported history of cancer at risk assessment and 609 lacking follow-up vital status. Race and ethnicity were available via self-report and cancer registries. We excluded 1,457 women with discrepant race and ethnicity information from these two sources, leaving 37,939 women for analysis. Each registry and the SCC received institutional review board approval for active or passive consenting processes or a waiver of consent to enroll participants, link data, and perform analytic studies. All procedures are Health Insurance Portability and Accountability Act (HIPAA) compliant and the registries and the SCC received a Federal Certificate of Confidentiality and other protection for the identities of women, physicians, and facilities involved in this research.

### Risk Prediction Models and Covariates

BCSC registries collected similar questionnaire data at each mammography examination, including demographic information, zip code of residence, height, weight, reproductive history and menopausal hormone use. Income was defined as annual median values for zip codes of residence based on 2000 census data.

Radiologists recorded breast density, assessed visually, according to the Breast Imaging Reporting and Data System (BI-RADS) in categories of increasing risk: a) almost entirely fat; b) scattered fibroglandular densities; c) heterogeneously dense and d) extremely dense.[26]

Visits at which records indicated that a mammogram was performed for screening, and which were not preceded by a mammogram within 9 months, were classified as screening visits. We estimated 5-year absolute breast cancer risk using questionnaire data (and BI-RADS density when included within a risk model) collected at the last screening visit prior to diagnosis or if unavailable, at another pre-diagnostic visit. We preferentially used risk factor data from the last screening visit 1–2 years prior to breast cancer diagnosis (n = 12,749) or if unavailable, at a screening visit performed 2–5 years prior to diagnosis (n = 10,935). Otherwise, we used data collected at the screening visit closest to the date of diagnosis within the interval from within one year prior to 30 days post-diagnosis (n = 6,248). When questionnaires were unavailable for these visits, we used information collected with any mammogram during these periods using the same prioritization: 1–2 years prior to diagnosis (n = 1,001), 2–5 years prior to diagnosis (n = 827) and one year prior to 30 days post-diagnosis (n = 6,179).

We estimated absolute 5-year risk of developing breast cancer according to the BCSC-1 model[8] (which includes separate algorithms for pre- and postmenopausal women), BCRAT[9, 10, 14] and BCSC-5[5] using factors specified in S1 Table. We considered 5 years as a clinically relevant interval over which to predict risk as suggested in American Society of Clinical Oncology guidelines.[27] For BCSC-1, which estimates breast cancer risk within 1 year of a screening mammogram, we estimated five-year risk as [1 –(1 −probability of invasive cancer in 1 year)^5^] as previously reported.[8] Of 37,939 women included in this study, 6% are excluded in BCSC-1 analyses because of missing menopausal status and 32% are excluded in BCSC-5 analyses due to missing mammographic density. Missing data for other risk factors were handled as specified by the models; BCSC-1: missing values were modeled; BCRAT: the lowest risk value is used, and BCSC-5: missing values for prior biopsy and family history were allowed and assumed not to alter risk. Missing data was generally encountered when a factor was not collected by a breast imaging facility. We performed sensitivity analyses restricted to women without missing data to assess potential bias.

Mode of breast cancer detection was categorized as screen-detected, interval, or clinically-detected. Mammogram BI-RADS assessment codes of 1 or 2 or 3 with recommendations for routine or short interval follow-up were classified as negative; results of 0, 4 or 5 or 3 with recommendation for immediate evaluation were classified as positive. Screen-detected cancers were defined as tumors diagnosed within one year of a positive screening mammogram; those preceded by a negative screening mammogram within one year were considered interval cancers. Cancers were defined as clinically-detected if associated with a diagnostic mammogram not preceded by a screening mammogram within a year. Cases not meeting these criteria or lacking mammogram data within a year of diagnosis were considered mode of detection missing.

### Breast cancer pathology

Breast cancer size, histological grade, estrogen receptor (ER) status, lymph node status and American Joint Committee on Cancer Collaborative Stage (5^th^ edition) were collected. Treatment data included surgical procedure and administration of adjuvant therapy (yes/no).

### Vital Status

We collected vital status information, including date of last ascertainment. Among deceased women, cause of death was obtained from cancer registries (preferred source) or state records. Deaths were ascertained through 2008 or 2009, depending on registry. Women without death records were presumed to be alive through the last date of ascertainment.

### Analysis

Absolute 5-year risks of developing breast cancer were estimated per model and grouped categorically: <1.0%; 1.0–1.66%; and ≥ 1.67% (eligibility threshold for chemoprevention trials[2]). To assess factors underlying relationships between predicted risk of developing breast cancer and mortality, we present descriptive data in which predicted risk of developing breast cancer is related to tumor size, grade, ER status, axillary lymph node status, stage and mode of presentation.

We computed hazard ratios (HRs) and 95% confidence intervals (CIs) for risk of breast cancer death and death from unrelated causes after breast cancer diagnosis using Cox proportional hazards models. Proportional hazards assumptions were checked by examination of marginal survival plots.

In unadjusted analyses, we compared breast cancer mortality (and mortality secondary to other causes) in each of the two highest categories of predicted risk of developing breast cancer to those in the lowest category (1.0% — 1.66% versus <1.0% and ≥1.67% versus <1.0%). Given that breast cancer death may show a non-linear dependence on age, we fit separate Cox models by categories of age of diagnosis: 35–44, 45–54, 55–69 and 70–89 years. Since the models predict risk of developing breast cancer, rather than risk of developing fatal breast cancer, we performed additional analyses to assess if observed associations are independent of factors related to prognosis that are not included in the models. We also assessed the association of the model predictions with risk of non-breast cancer death (i.e. competing risks). Of possible factors, age is most critical, because breast cancers among older women are generally less aggressive and these patients are more likely to die of unrelated causes. [28] Accordingly, models were adjusted for continuous age, income, registry, the year of diagnosis, mode of detection, AJCC stage and treatment.

Analyses were conducted using SAS software, version 9.2 (Cary, North Carolina).

## Results

### Estimates of Risk of Developing Breast Cancer Prior to Diagnosis by Model

**Fig 1:**
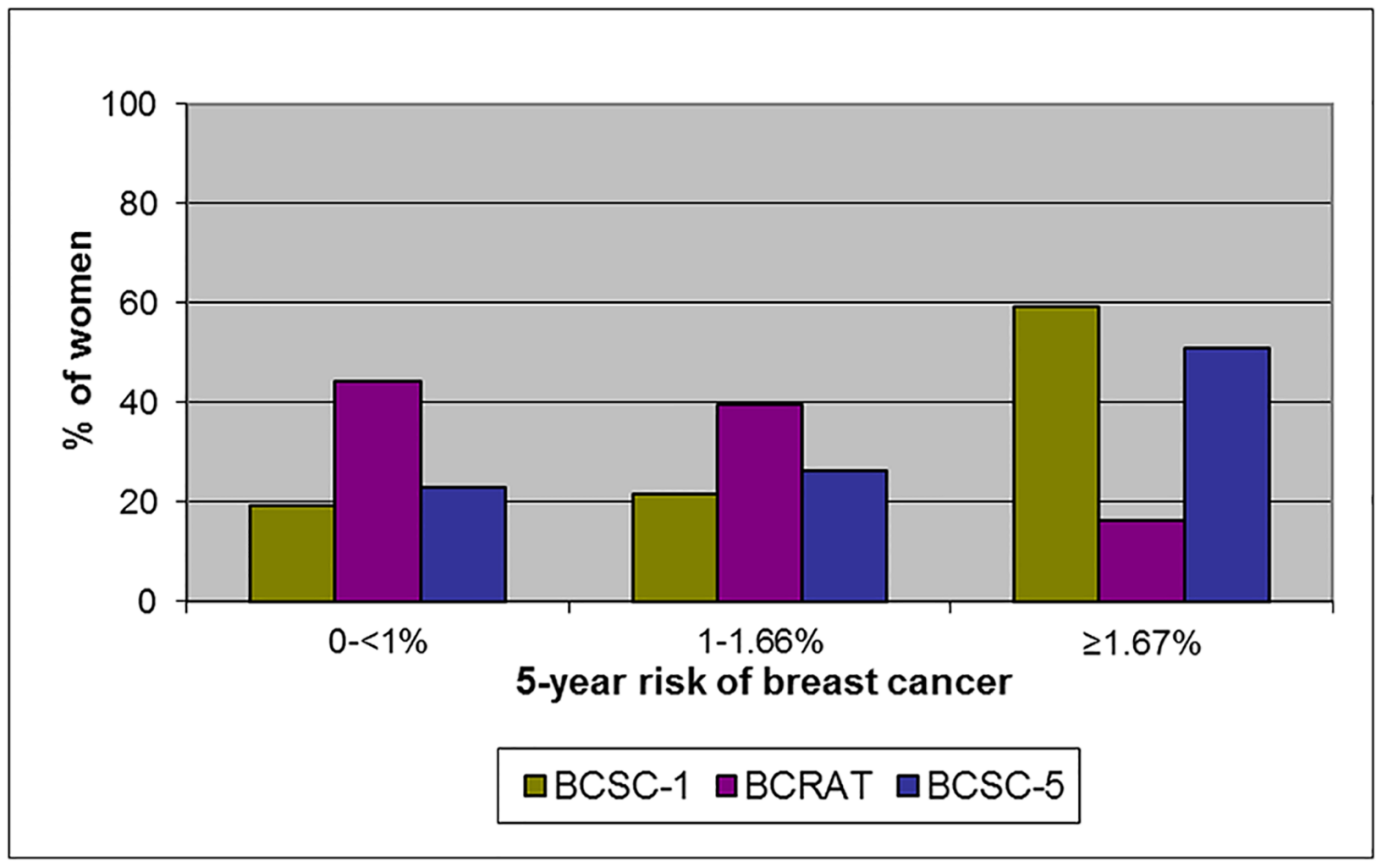
Categories of predicted risk of developing breast cancer.

Patient and tumor characteristics are presented in Table 1. The mean age of women was 58.6 (SD 12.2) years at time of risk assessment and 60.2 (SD 12.2) years at diagnosis. Models assigned different percentages of women to the highest stratum of predicted risk (≥1.67%); BCSC-1: 59.4%; BCRAT: 16.1% and BCSC-5: 50.9% (Fig 1). The three models varied with regard to percentages of women missing one or more included factor(s): BCSC-1: 78%, BCRAT: 82%, and BCSC-5: 25%. Among women without missing factors, the percentage of women in the highest risk category increased for BCRAT to 38.3% and decreased for BCSC-1 to 42.6%, which made risk distributions more similar among models (S1 Fig).

**Table 1:** Characteristics of 37,939 Women with Breast Cancer.

Characteristic	Category	N	(%)
***Patient characteristics at time of risk assessment (index mammogram)*:**			
Age at exam, years	35–49	10,209	(27)
	50–59	10,618	(28)
	60–69	8,502	(22)
	70–84	8,610	(23)
	Mean (s.d.) = 58.6 (12.2)		
Race/ethnicity	White	31,159	(82)
	African American	2,254	(6)
	Hispanic	2,557	(7)
	Native American	265	(1)
	Asian	1,704	(4)
Type and time of exam prior to diagnosis	Screen 1-2y	12,749	(34)
	Screen 2-5y	10,935	(29)
	Screen <1y	6,248	(16)
	Any mammogram 1-2y	1,001	(3)
	Any mammogram 2-5y	827	(2)
	Any mammogram <1y	6,179	(16)
BI-RADS breast density	Almost entirely fat	1,398	(5)
	Scattered	10,751	(41)
	Heterogeneously dense	11,383	(44)
	Extremely dense	2,553	(10)
	*Missing*^1^	*11*,*854*	
Family history of breast cancer	Yes	5,413	(19)
	No	22,562	(81)
	*Missing*	*9*,*964*	
Prior breast biopsy	Yes	7,273	(23)
	No	24,272	(77)
	*Missing*	*6*,*394*	
Age at menarche, years	≤11	1,355	(15)
	12–13	5,075	(57)
	≥14	2,526	(28)
	*Missing*^2^	*28*,*983*	
Age at first birth, years	<20	2,540	(12)
	20–24	4,422	(21)
	25–29	2,512	(12)
	<30 NOS	4,500	(22)
	Nulliparous or ≥30	6,887	(33)
	*Missing*	*17*,*078*	
Body mass index, kg/m^2^	<30 (non-obese)	13,096	(77)
	≥30 (obese)	3,918	(23)
	*Missing*	*20*,*925*	
Current HT use	Yes	8,157	(28)
	No	21,158	(72)
	*Missing*	*8*,*624*	
Prior breast procedure	Yes	6,758	(21)
	No	24,892	(79)
	*Missing*	*6*,*289*	
Surgical menopause	Yes	6,094	(25)
	No	18,542	(75)
	*Missing*	*13*,*303*	
Last mammogram result	Negative	18,335	(96)
	Positive	802	(4)
	*Missing*	*18*,*802*	
Annual median income (based on zip code)	<$43,000	9,353	(25)
	$43,000-<52,500	9,412	(25)
	$52,500-<68,000	9,239	(25)
	$68,000+	9,131	(25)
	*Missing*	*804*	
***Tumor characteristics***			
Year of diagnosis	1996–1998	7,718	(20)
	1999–2001	10,825	(29)
	2002–2004	10,599	(28)
	2005–2007	8,797	(23)
AJCC stage	I	19,148	(53)
	II	12,472	(34)
	III	3,845	(11)
	IV	888	(2)
	*Missing*	*1*,*586*	
Mode of detection	Screen detected	14,893	(54)
	Interval cancer	3,665	(13)
	Clinically detected	9,077	(33)
	*Missing*^3^	*10*,*304*	
Tumor size, cm	≤2	24,358	(68)
	>2	11,685	(32)
	*Missing*	*1*,*896*	
Positive lymph nodes	Yes	11,819	(31)
	No	26,120	(69)
Grade	1	8,273	(24)
	2	14,181	(41)
	3	11,879	(35)
	*Missing*	*3*,*606*	
Estrogen receptor status	Positive or borderline	25,112	(80)
	Negative	6,153	(20)
	*Missing*	*6*,*674*	
Progesterone receptor status	Positive or borderline	21,794	(71)
	Negative	8,896	(29)
	*Missing*	*7*,*249*	
Surgery	None	1,315	(4)
	Lumpectomy	21,699	(59)
	Mastectomy	14,078	(38)
	*Missing*	*847*	
Radiation	Yes	19,124	(51)
	No	18,494	(49)
	*Missing*	*321*	
Chemotherapy	Yes	13,442	(37)
	No	23,152	(63)
	*Missing*	*1*,*345*	

^1^ N = 2,790 women with breast density on different scale than BI-RADS.

^2^ N = 4,494 women with age at menarche ≤12, cannot distinguish between age ≤11 and 12–13.

^3^ N = 6,976 women with no mammogram in prior year; N = 177 women missing result on screening mammogram; and N = 3,151 women with prior mammogram but indication is not screening or evaluation of breast problem.

Abbreviation: HT hormone replacement.

Outcomes over a median of more than 5 years of follow-up (range per model 5.4–5.6 years) included: BCRAT: 6,021 total deaths, with 2,993 (49.7%) related to breast cancer; BCSC-1: 5,742 total deaths, with 2,785 (48.5%) related to breast cancer and BCSC-5: 3,859 deaths, with 1,910 (49.5%) related to breast cancer.

### Predicted Risk of Developing Breast Cancer Versus Clinicopathologic Characteristics of Tumors

**Fig 2:**
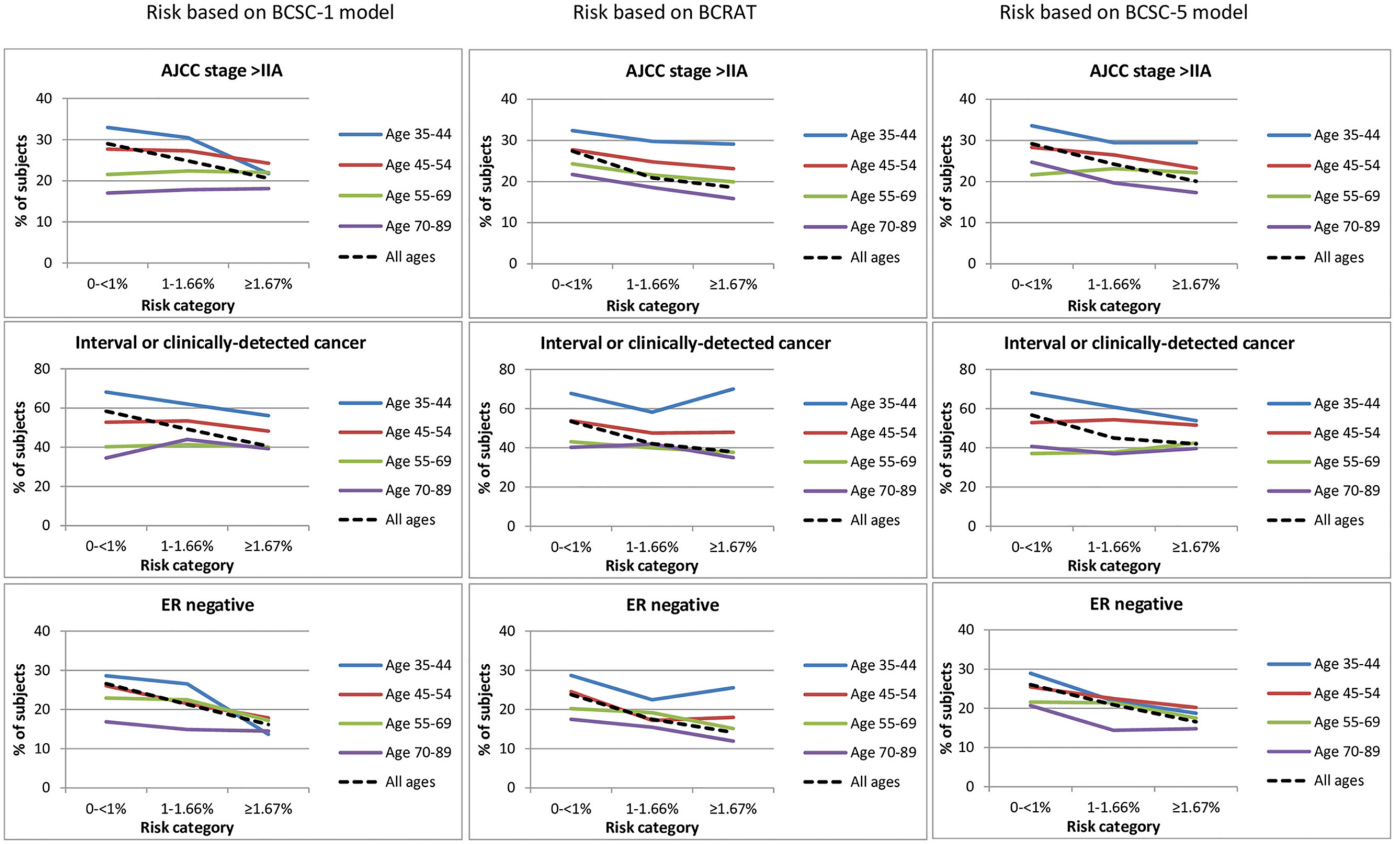
Predicted 5-year risk of developing breast cancer versus clinicopathologic characteristics.

For descriptive purposes, we visually assessed relationships of patterns of predicted risk of developing breast cancer and selected clinicopathological tumor features (Fig 2; S2 and S3 Tables). Increasing predicted risk of developing breast cancer appeared to be inversely related to percentage of tumors with adverse prognostic features, including stage >IIA, interval or clinical presentation and ER-negative status; similar inverse associations were found for tumor size >2 cm, histologic grade 3 and positive axillary lymph nodes (data not shown). Associations were generally more evident among younger women.

### Overall Association of Predicted Breast Cancer Risk with Cause Specific Mortality

**Fig 3:**
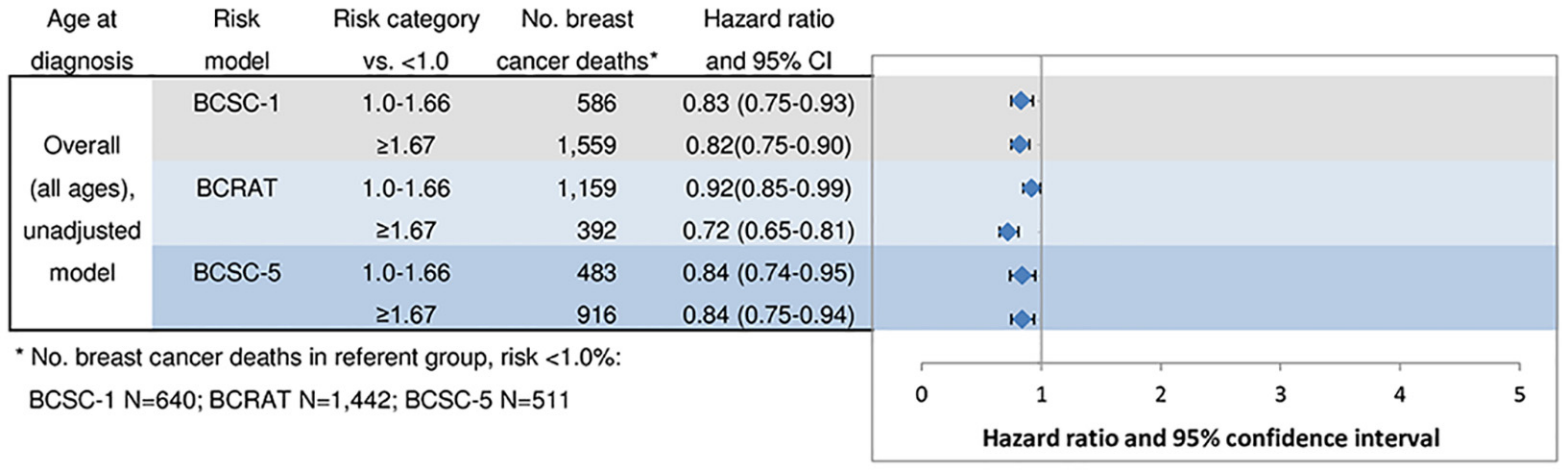
Predicted risk of developing breast cancer versus risk of death (unadjusted).

In unadjusted analyses, reflecting the application of models as developed, and including women with missing factors specified in models, women with the highest predicted risk of developing breast cancer (≥ 1.67%) were at significantly lower risk of breast cancer death than those at lowest predicted risk (<1.0%): BCSC-1: HR = 0.82 (95% CI = 0.75–0.90); BCRAT: HR = 0.72 (95% CI = 0.65–0.81) and BCSC-5: HR = 0.84 (95% CI = 0.75–0.94) (Fig 3). Analyses of women with complete risk factor information specified per model, yielded similar results, although estimates for BCRAT were not statistically significant (S2 Fig). In all unadjusted analyses of the three risk prediction models, women with greater predicted risk of developing breast cancer had a higher risk of non-breast cancer death (S3 Fig); results were similar for women with complete risk factor information as specified by the models (S4 Fig).

### Relationship of Predicted Breast Cancer Risk to Cause-Specific Mortality by Age Group

**Fig 4:**
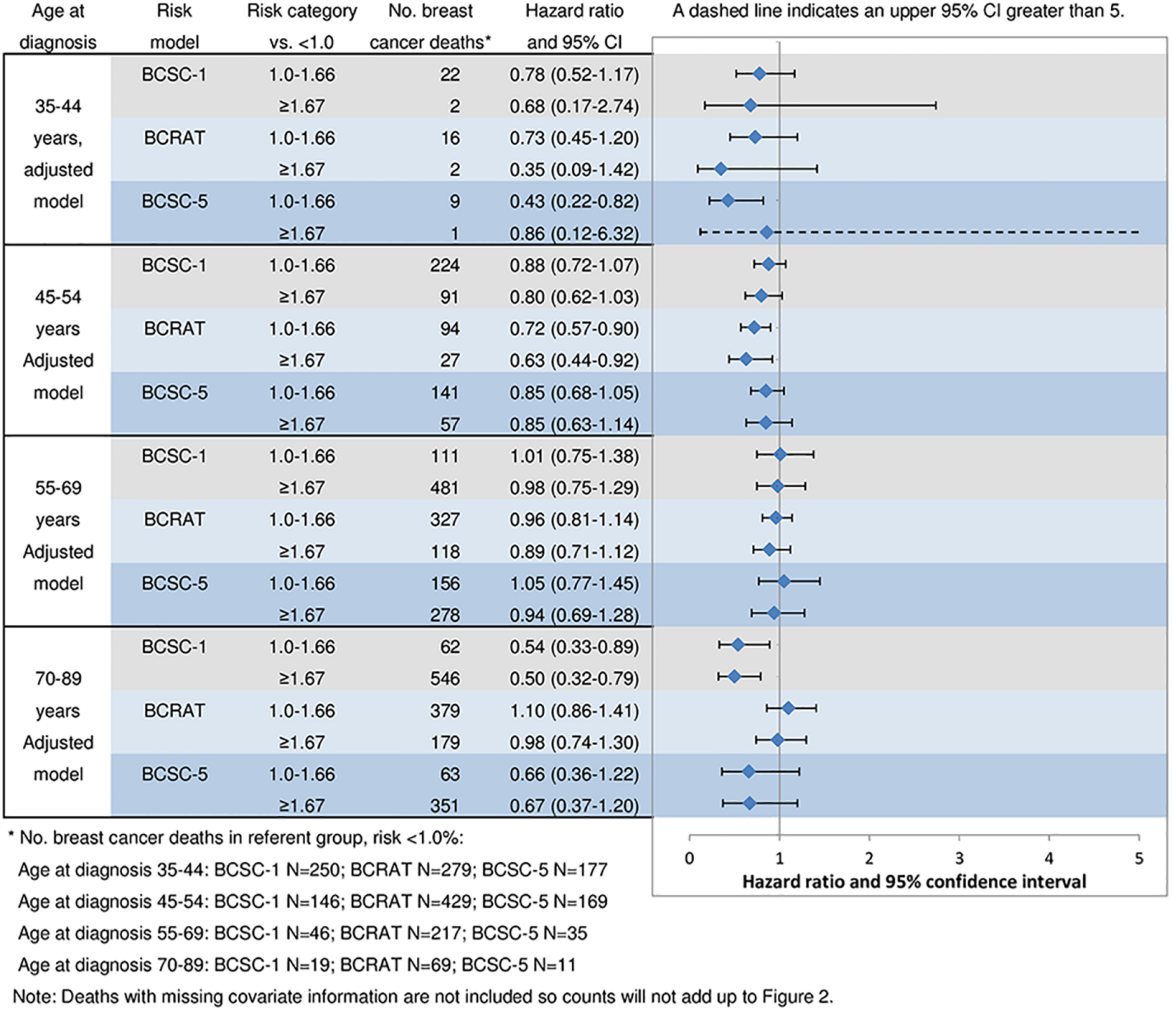
Predicted risk of developing breast cancer versus risk of death (age-stratified, adjusted models). Age-stratified models, adjusted for age in single years, registry, year of diagnosis, mode of detection, AJCC stage, treatment (surgery and chemotherapy: yes/no) and income (zip code of residence)

Relationships of predicted risk of developing breast cancer were compared to breast cancer mortality by risk model, stratified by age at diagnosis and adjusted for continuous age, income, registry, year of diagnosis, mode of detection, stage and treatment (Fig 4). None of the groups with higher risks of developing breast cancer (1.0–1.66% or ≥1.67%) had a statistically significant increased risk of breast cancer death, compared to the lowest risk categories (<1.0%). A higher predicted risk of developing breast cancer was associated with a statistically significant lower risk of breast cancer death among cases for several comparisons: 1) BCSC-1: women aged ≥70 years (for risk category 1.0–1.66%: HR = 0.54, 95% CI = 0.33–0.89; for risk ≥1.67%: HR = 0.50, 95% CI = 0.32–0.79); 2) BCRAT: women aged 45–54 years (for risk category 1.0–1.66%: HR = 0.72, 95% CI = 0.57–0.90; for risk ≥1.67%: HR = 0.63, 95% CI = 0.44–0.92) and 3) BCSC-5: women ages 35–44 years (for risk category 1.0–1.66%: HR = 0.43, 95% CI = 0.22–0.82). Analyses limited to women with complete risk factor information specified by models yielded similar results (S5 Fig).

Risk of developing breast cancer was not related to risk of non-breast cancer death in age stratified and adjusted models (S6 and S7 Figs).

## Discussion

This analysis evaluates whether risks of developing incident breast cancer, as assessed with three models, is related to risk of death among cases. These data have implications for both clinical practice and research.

Clinically, the discussion of a new breast cancer diagnosis is stressful for both patients and clinicians, especially if a patient has received prior information about risk of developing breast cancer and used this information to decide about participating in screening and preventive interventions [29]. Our analysis clarifies the relationship of risk estimates of developing breast cancer and the pathologic features and prognosis of cancers diagnosed. Second, from a research perspective, our data inform the utility of using three risk prediction models for planning trials that aim to test interventions to reduce breast cancer mortality, as opposed to breast cancer incidence. Accordingly, in planning studies evaluating screening, chemoprevention or lifestyle modification, it may be important to achieve the statistical power required to assess a mortality benefit, which is linked to both the risk of incident breast cancer and the prognosis of such tumors. The efficiency of such studies would be increased by recruiting women who are at increased risk of developing fatal tumors, if such models could be developed.

Our case-only analysis demonstrates that the predicted risk of developing breast cancer, as estimated with any of three models used in the U.S., was inversely associated with risk of breast cancer death among cases, over a median follow-up of more than 5 years post-diagnosis. Consistent with these results, increased risk of developing breast cancer was associated with better prognostic features related to mode of detection, tumor size, grade, stage and ER status. Unadjusted analyses were based only on the risk scores generated with the models as originally developed. However, in evaluating mortality, we used adjustments for additional factors. With increasing age, breast cancer incidence increases, prognosis generally improves, and non-breast cancer deaths increase [28]. Thus, to account for these age-related associations, we performed age-stratified analyses, further adjusted for continuous age. In addition, to exclude confounding by mode of detection, stage and treatment, we also adjusted for these factors. These analyses generally revealed non-significant inverse associations between higher predicted risk of developing breast cancer and risk of breast cancer death among cases, although significant associations were found for some subgroups. In contrast, accounting for age eliminated associations between predicted risk of developing breast cancer and competing risks of death. Therefore, the observed inverse associations between risk of developing breast cancer and disease specific mortality among cases do not seem to be entirely explained by known prognostic factors or competing risks. Thus, a low predicted risk of developing breast cancer does not portend an excellent prognosis once the cancer is diagnosed, and a high predicted risk does not imply tumor aggressiveness.

Tools to identify women at increased risk of fatal breast cancers would facilitate prevention studies aimed at reducing mortality by guiding specification of inclusion criteria and determination of sample sizes. In the IBIS-1 trial, which employed a risk assessment tool not included herein based on family history and prior diagnosis of benign breast disease, administration of 20 mg of tamoxifen daily reduced breast cancer incidence by 29% compared with placebo. Nonetheless, a mortality benefit was not demonstrated, most likely (as the authors note) because the analysis was severely underpowered.[30] Although current models used to plan prevention trials predict total breast cancer incidence, rather than risk of fatal breast cancers, consideration of tumor subtype and prognosis in relation to predicted risk may help guide future model development.

Developing models to predict clinically aggressive forms of breast cancer will be challenging, and was not the intent of this report. The development of a model to predict absolute risk of developing fatal breast cancer, would necessitate modeling of both the risk of incident cancer and the prognosis of such cancers. There is considerable evidence that breast cancer risk factors vary by tumor subtype and that different subtypes vary in clinical behavior, suggesting links between risk and tumor aggressiveness. [17] Given that several breast cancer risk factor associations differ by molecular tumor subtypes (e.g. ER-positive versus ER-negative basal breast cancers) with differences in prognoses, subtype-specific models may be needed to predict risk of lethal subtypes.[13, 15, 16, 31] Further, investigations, albeit some preliminary, suggest that circulating sex-steroid levels and analyses of benign breast tissues may identify markers that predict breast cancer risk overall, and potentially by subtype or clinical behavior [32–38]. It is likely that improving prediction models will require inclusion of additional known risk factors and novel measures, potentially including circulating factors, genetic markers, molecular imaging and even tissue sampling of high-risk women. For example, a preliminary study of women with early stage breast cancer links inflammation in white adipose tissue with increased circulating markers of inflammation, metabolic syndrome and reduced distant recurrence-free survival [39].

Strengths of this study include the large database with detailed information about screening, prospectively collected risk information and pathological outcomes. Although some analyses were limited by missing data, this largely reflected the scope of the questionnaires employed at different registries, rather than systematic biases among individuals; sensitivity analyses restricted to women with complete data did not change overall conclusions. Although HRs were adjusted for treatment in broad categories, treatment would only confound associations if women with high risk scores, as evaluated prior to diagnosis, received less effective treatment. Attribution of cause of death may be misclassified, but unless non-random and frequent, it would be unlikely to alter our conclusions. Although ER-positive cancers may recur more than five years post-diagnosis, our power to assess these endpoints was limited. Finally, our conclusions are restricted to the three models evaluated, and are based on relating risk of developing cancer to fatality, only among cases.

In summary, this analysis demonstrates that higher predicted risk of developing breast cancer, was not associated with higher risk of breast cancer mortality among cases, and was possibly related to lower mortality among some women with breast cancer. This information may be important to communicate to women who have received information about risk of developing cancer in the past and are now receiving a new breast cancer diagnosis. In addition, the development of models to predict risk of potentially fatal breast cancers could facilitate efficient planning of research to reduce breast cancer deaths through targeted early detection or prevention.

## Supporting Information

S1 Fig 5-year predicted risk of developing breast cancer (subjects with complete risk factor information). (DOCX)

S2 Fig 5-year predicted risk of developing breast cancer versus breast cancer death (subjects with complete risk factor information). (DOCX)

S3 Fig 5-year predicted risk of developing breast cancer versus non-breast cancer mortality. (DOCX)

S4 Fig 5-year predicted risk of developing breast cancer versus non-breast cancer mortality (subjects with complete risk factor information). (DOCX)

S5 Fig 5-year predicted risk of developing breast cancer and breast cancer mortality (subjects with complete risk factor information). (DOCX)

S6 Fig 5-year predicted risk of developing breast cancer and non-breast cancer mortality. (DOCX)

S7 Fig 5-year predicted risk of developing breast cancer and non-breast cancer mortality (adjusted, subjects with complete risk factor information). (DOCX)

S1 Table Factors included in breast cancer risk prediction models. (DOCX)

S2 Table Tumor characteristics by 5-year predicted risk of developing breast cancer. (DOCX)

S3 Table Tumor characteristics by 5-year predicted risk of developing breast cancer (subjects with complete risk factors). (DOCX)
