# Supplementary material for: Relationship of Predicted Risk of Developing Invasive Breast Cancer, as Assessed with Three Models, and Breast Cancer Mortality among Breast Cancer Patients
Source: PLoS One. 2016 Aug 25;11(8):e0160966. doi: 10.1371/journal.pone.0160966 (PMC4999085; doi:10.1371/journal.pone.0160966)
Supplement: S1 Table — (DOCX) [file pone.0160966.s008.docx]

|  | **BCSC-1 ^(17)^** | **BCSC-1 ^(17)^** |  |  |
| --- | --- | --- | --- | --- |
| **Risk factor** | pre-menopausal | post-menopausal | **BCRAT^(18,19)^** | **BCSC-5 ^(14)^** |
| Age | X | X | X | X |
| Race/ethnicity |  | X | X | X |
| Family history of breast cancer | X | X | X | X |
| Biopsy history |  |  | X | X |
| Atypical hyperplasia |  |  | X |  |
| Any breast procedure | X | X |  |  |
| Age at first birth |  | X | X |  |
| Age at menarche |  |  | X |  |
| BI-RADS breast density | X | X |  | X |
| Body mass index |  | X |  |  |
| Current hormone therapy use |  | X |  |  |
| Surgical menopause |  | X |  |  |
| Last mammogram result |  | X |  |  |

References for risk models:

^17^Barlow WE, White E, Ballard-Barbash R, et al: Prospective breast cancer risk prediction model for women undergoing screening mammography. J Natl Cancer Inst 98:1204-14, 2006

^18^Costantino J, Gial MH, Pee D, et al: Validation studies for models to project the risk of invasive and total breast cancer. J Natl Cancer Inst 91:1541-8, 1999

^19^Gail MH, Costantino JP, Pee D, et al: Projecting individualized absolute invasive breast cancer risk in African American women. J Natl Cancer Inst 99:1782-92, 2007

^22^Matsuno RK, Costantino JP, Ziegler RG, et al: Projecting individualized absolute invasive breast cancer risk in asian and pacific islander american women. J Natl Cancer Inst 103:951-61, 2011

^14^Tice JA, Cummings SR, Smith-Bindman R, et al. Using clinical factors and mammographic breast density to estimate breast cancer risk: development and validation of a new predictive model. Ann Intern Med 148(5):337-47, 2008
