# Supplementary material for: Relationship of Predicted Risk of Developing Invasive Breast Cancer, as Assessed with Three Models, and Breast Cancer Mortality among Breast Cancer Patients
Source: PLoS One. 2016 Aug 25;11(8):e0160966. doi: 10.1371/journal.pone.0160966 (PMC4999085; doi:10.1371/journal.pone.0160966)
Supplement: Supplementary file 2 [file pone.0160966.s001.docx]

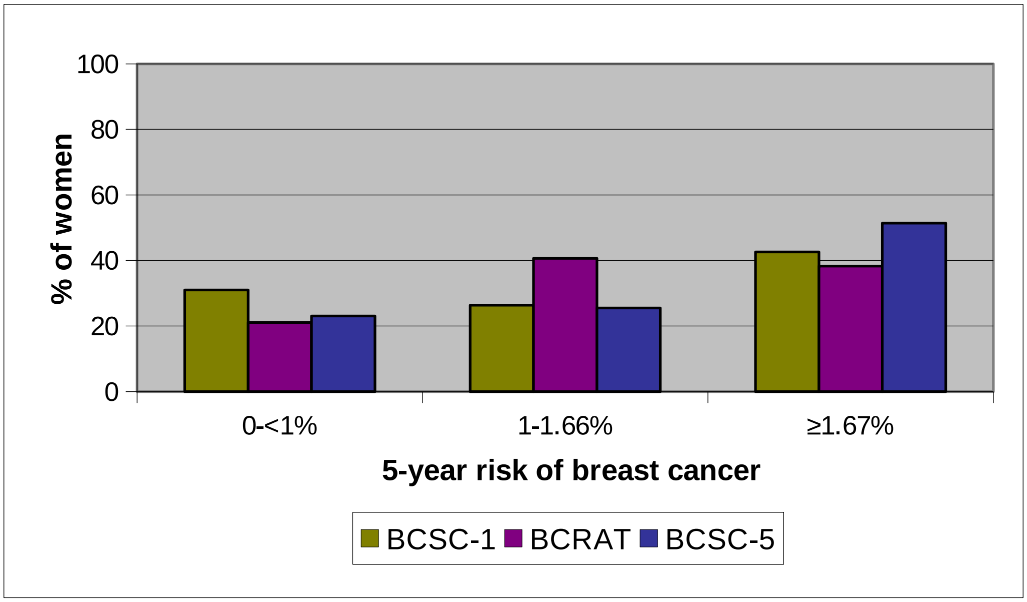
100
80
60
40
20
0
% of women
0-<1%
1-1.66%
≥1.67%
5-year risk of breast cancer
BCSC-1
BCRAT
BCSC-5
