# Supplementary material for: Relationship of Predicted Risk of Developing Invasive Breast Cancer, as Assessed with Three Models, and Breast Cancer Mortality among Breast Cancer Patients
Source: PLoS One. 2016 Aug 25;11(8):e0160966. doi: 10.1371/journal.pone.0160966 (PMC4999085; doi:10.1371/journal.pone.0160966)
Supplement: Supplementary file 3 [file pone.0160966.s002.docx]

Age at diagnosis	Risk model	Risk category vs. <1.0	No. breast cancer deaths*	Hazard ratio and 95% CI
Overall (all ages), unadjusted model	BCSC-1	1.0-1.66	121	0.74 (0.59-0.93)
		≥1.67	192	0.73 (0.59-0.89)
	BCRAT	1.0-1.66	162	0.86 (0.67-1.10)
		≥1.67	153	0.92 (0.72-1.18)
	BCSC-5	1.0-1.66	313	0.79 (0.68-0.91)
		≥1.67	647	0.83 (0.73-0.94)

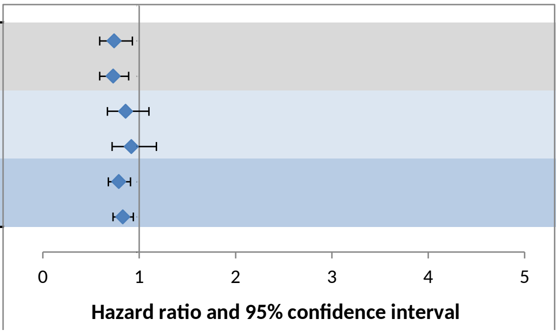

* No. breast cancer deaths in referent group, risk <1.0%:
BCSC-1 N=187; BCRAT N=103; BCSC-5 N=367
