# Supplementary material for: Relationship of Predicted Risk of Developing Invasive Breast Cancer, as Assessed with Three Models, and Breast Cancer Mortality among Breast Cancer Patients
Source: PLoS One. 2016 Aug 25;11(8):e0160966. doi: 10.1371/journal.pone.0160966 (PMC4999085; doi:10.1371/journal.pone.0160966)
Supplement: Supplementary file 4 [file pone.0160966.s003.docx]

Age at diagnosis	Risk model	Risk category vs. <1.0	No. non-breast cancer deaths*	Hazard ratio and 95% CI
All ages Unadjusted model	BCSC-1	1.0-1.66	441	2.10 (1.77-2.49)
		≥1.67	2,324	4.10 (3.54-4.75)
	BCRAT	1.0-1.66	1,680	2.93 (2.67-3.20)
		≥1.67	523	2.80 (2.51-3.11)
	BCSC-5	1.0-1.66	430	2.33 (1.95-2.79)
		≥1.67	1,356	3.93 (3.34-4.62)

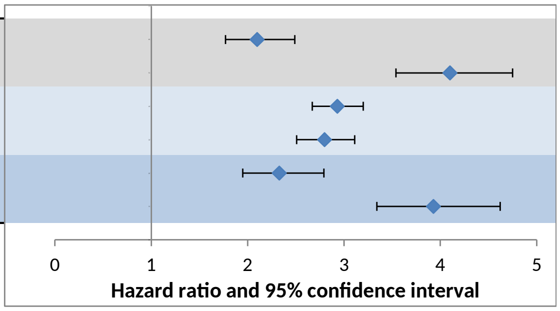

* No. non-breast cancer deaths in referent group, risk <1.0%:
BCSC-1 N=192; BCRAT N=655; BCSC-5 N=163
