# Supplementary material for: Relationship of Predicted Risk of Developing Invasive Breast Cancer, as Assessed with Three Models, and Breast Cancer Mortality among Breast Cancer Patients
Source: PLoS One. 2016 Aug 25;11(8):e0160966. doi: 10.1371/journal.pone.0160966 (PMC4999085; doi:10.1371/journal.pone.0160966)
Supplement: Supplementary file 5 [file pone.0160966.s004.docx]

Age at diagnosis	Risk model	Risk category vs. <1.0	No. non-breast cancer deaths*	Hazard ratio and 95% CI
All ages Unadjusted model	BCSC-1	1.0-1.66	50	1.39 (0.92-2.10)
		≥1.67	219	3.84 (2.75-5.36)
	BCRAT	1.0-1.66	208	3.27 (2.29-4.65)
		≥1.67	253	4.57 (3.22-6.48)
	BCSC-5	1.0-1.66	302	2.44 (1.97-3.03)
		≥1.67	991	4.10 (3.38-4.98)

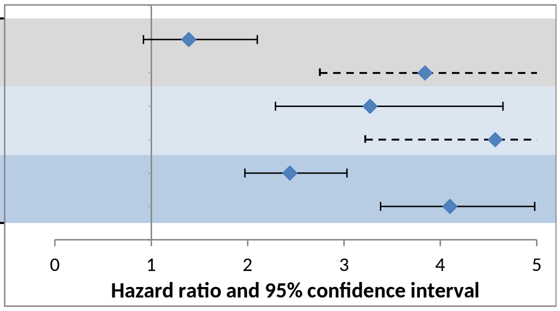

* No. non-breast cancer deaths in referent group, risk <1.0%:
BCSC-1 N=41; BCRAT N=36; BCSC-5 N=114
