# Supplementary material for: Relationship of Predicted Risk of Developing Invasive Breast Cancer, as Assessed with Three Models, and Breast Cancer Mortality among Breast Cancer Patients
Source: PLoS One. 2016 Aug 25;11(8):e0160966. doi: 10.1371/journal.pone.0160966 (PMC4999085; doi:10.1371/journal.pone.0160966)
Supplement: Supplementary file 6 [file pone.0160966.s005.docx]

Age at diagnosis	Risk model	Risk category vs. <1.0	No. breast cancer deaths*	Hazard ratio and 95% CI
35-44 years, adjusted model	BCSC-1	1.0-1.66	12	0.59 (0.33-1.05)
		≥1.67	2	1.09 (0.26-4.52)
	BCRAT	1.0-1.66	2	0.23 (0.05-0.97)
		≥1.67	1	0.27 (0.03-2.46)
	BCSC-5	1.0-1.66	7	0.38 (0.18-0.78)
		≥1.67	1	0.89 (0.12-6.52)
45-54 years Adjusted model	BCSC-1	1.0-1.66	71	0.89 (0.62-1.28)
		≥1.67	32	0.85 (0.55-1.31)
	BCRAT	1.0-1.66	36	0.87 (0.56-1.35)
		≥1.67	14	0.89 (0.48-1.63)
	BCSC-5	1.0-1.66	94	0.80 (0.61-1.04)
		≥1.67	45	0.86 (0.62-1.19)
55-69 years Adjusted model	BCSC-1	1.0-1.66	14	3.76 (0.49-28.7)
		≥1.67	63	2.22 (0.30-16.6)
	BCRAT	1.0-1.66	59	1.28 (0.65-2.51)
		≥1.67	50	1.41 (0.70-2.83)
	BCSC-5	1.0-1.66	108	1.11 (0.77-1.60)
		≥1.67	205	0.98 (0.69-1.40)
70-89 years Adjusted model	BCSC-1	1.0-1.66	6	0.67 (0.08-5.75)
		≥1.67	60	0.42 (0.06-3.14)
	BCRAT	1.0-1.66	41	Not enough
		≥1.67	68	data
	BCSC-5	1.0-1.66	43	0.52 (0.26-1.05)
		≥1.67	253	0.59 (0.30-1.13)

A dashed line indicates an upper 95% CI greater than 5.

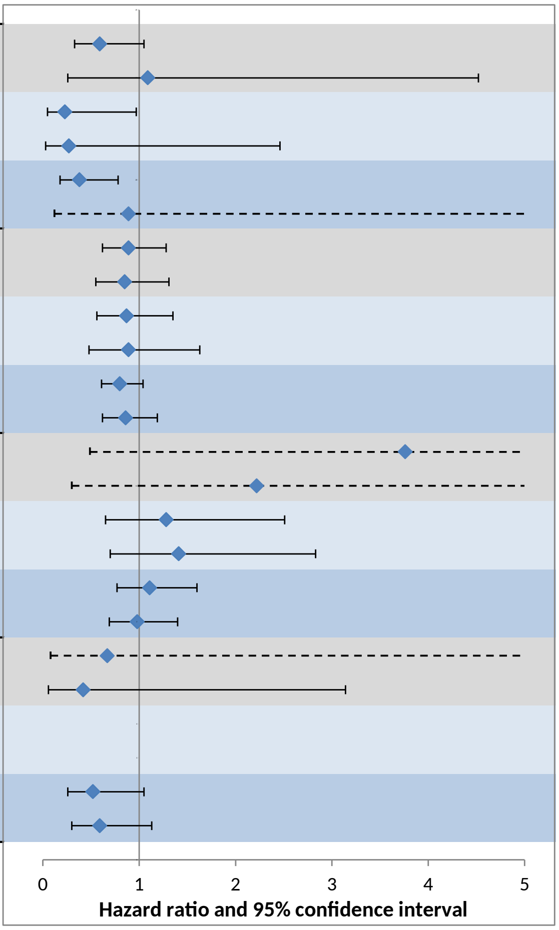

* No. breast cancer deaths in referent group, risk <1.0%:

Age at diagnosis 35-44: BCSC-1 N=100; BCRAT N=36; BCSC-5 N=129

Age at diagnosis 45-54: BCSC-1 N=46; BCRAT N=37; BCSC-5 N=122

Age at diagnosis 55-69: BCSC-1 N=1; BCRAT N=9; BCSC-5 N=28

Age at diagnosis 70-89: BCSC-1 N=1; BCRAT N=0; BCSC-5 N=10

Note: Deaths with missing covariate information are not included so counts will not add up to Supplemental Figure 2.
