# Supplementary material for: Relationship of Predicted Risk of Developing Invasive Breast Cancer, as Assessed with Three Models, and Breast Cancer Mortality among Breast Cancer Patients
Source: PLoS One. 2016 Aug 25;11(8):e0160966. doi: 10.1371/journal.pone.0160966 (PMC4999085; doi:10.1371/journal.pone.0160966)
Supplement: Supplementary file 7 [file pone.0160966.s006.docx]

Age at diagnosis	Risk model	Risk category vs. <1.0	No. non-breast cancer deaths	Hazard ratio and 95% CI
35-44 years Adjusted model	BCSC-1	1.0-1.66	3	1.08 (0.37-3.18)
		≥1.67	1	3.27 (0.42-25.2)
	BCRAT	1.0-1.66	1	0.30 (0.04-2.30)
		≥1.67	0	Not enough data
	BCSC-5	1.0-1.66	1	0.24 (0.03-1.83)
		≥1.67	0	Not enough data
45-54 years Adjusted model	BCSC-1	1.0-1.66	57	0.98 (0.64-1.48)
		≥1.67	30	0.96 (0.58-1.59)
	BCRAT	1.0-1.66	25	0.67 (0.43-1.04)
		≥1.67	8	0.63 (0.31-1.26)
	BCSC-5	1.0-1.66	36	0.82 (0.53-1.25)
		≥1.67	18	0.96 (0.57-1.64)
55-69 years Adjusted model	BCSC-1	1.0-1.66	77	0.85 (0.62-1.18)
		≥1.67	437	0.72 (0.54-0.96)
	BCRAT	1.0-1.66	301	0.98 (0.80-1.19)
		≥1.67	130	0.96 (0.74-1.23)
	BCSC-5	1.0-1.66	102	0.80 (0.56-1.13)
		≥1.67	253	0.75 (0.54-1.06)
70-89 years Adjusted model	BCSC-1	1.0-1.66	154	0.80 (0.56-1.15)
		≥1.67	1,169	0.66 (0.47-0.92)
	BCRAT	1.0-1.66	787	0.95 (0.80-1.12)
		≥1.67	409	0.82 (0.68-0.99)
	BCSC-5	1.0-1.66	155	0.94 (0.60-1.48)
		≥1.67	710	0.74 (0.48-1.15)

A dashed line indicates an upper 95% CI greater than 5.

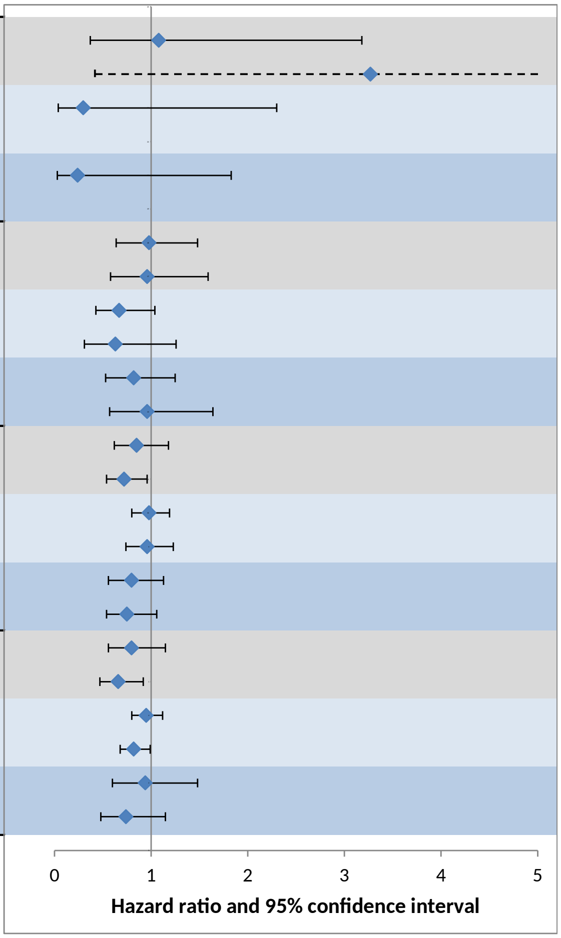

* No. non-breast cancer deaths in referent group, risk <1.0%:

Age at diagnosis 35-44: BCSC-1 N=25; BCRAT N=35; BCSC-5 N=25
Age at diagnosis 45-54: BCSC-1 N=31; BCRAT N=112; BCSC-5 N=43
Age at diagnosis 55-69: BCSC-1 N=39; BCRAT N=131; BCSC-5 N=31
Age at diagnosis 70-89: BCSC-1 N=24; BCRAT N=152; BCSC-5 N=20

Note: Deaths with missing covariate information are not included so counts will not add up to Supplemental Figure 4.
