# Supplementary material for: Relationship of Predicted Risk of Developing Invasive Breast Cancer, as Assessed with Three Models, and Breast Cancer Mortality among Breast Cancer Patients
Source: PLoS One. 2016 Aug 25;11(8):e0160966. doi: 10.1371/journal.pone.0160966 (PMC4999085; doi:10.1371/journal.pone.0160966)
Supplement: Supplementary file 8 [file pone.0160966.s007.docx]

Age at diagnosis	Risk model	Risk category vs. <1.0	No. non-breast cancer deaths	Hazard ratio and 95% CI
35-44 years Adjusted model	BCSC-1	1.0-1.66	2	0.74 (0.15-3.57)
		≥1.67	0	Not enough data
	BCRAT	1.0-1.66	1	1.54 (0.11-22.5)
		≥1.67	0	Not enough data
	BCSC-5	1.0-1.66	1	0.26 (0.03-2.11)
		≥1.67	0	Not enough data
45-54 years Adjusted model	BCSC-1	1.0-1.66	13	0.79 (0.35-1.77)
		≥1.67	7	0.84 (0.32-2.24)
	BCRAT	1.0-1.66	9	0.81 (0.31-2.09)
		≥1.67	7	1.46 (0.54-4.01)
	BCSC-5	1.0-1.66	22	0.79 (0.46-1.35)
		≥1.67	15	1.13 (0.62-2.06)
55-69 years Adjusted model	BCSC-1	1.0-1.66	10	0.63 (0.25-1.55)
		≥1.67	57	0.31 (0.13-0.72)
	BCRAT	1.0-1.66	58	0.70 (0.37-1.33)
		≥1.67	51	0.70 (0.36-1.36)
	BCSC-5	1.0-1.66	74	0.85 (0.58-1.26)
		≥1.67	208	0.79 (0.54-1.14)
70-89 years Adjusted model	BCSC-1	1.0-1.66	13	2.14 (0.28-16.3)
		≥1.67	117	1.33 (0.18-9.71)
	BCRAT	1.0-1.66	100	1.05 (0.32-3.40)
		≥1.67	150	0.86 (0.27-2.78)
	BCSC-5	1.0-1.66	120	1.18 (0.67-2.05)
		≥1.67	534	0.97 (0.53-1.56)

A dashed line indicates an upper 95% CI greater than 5.

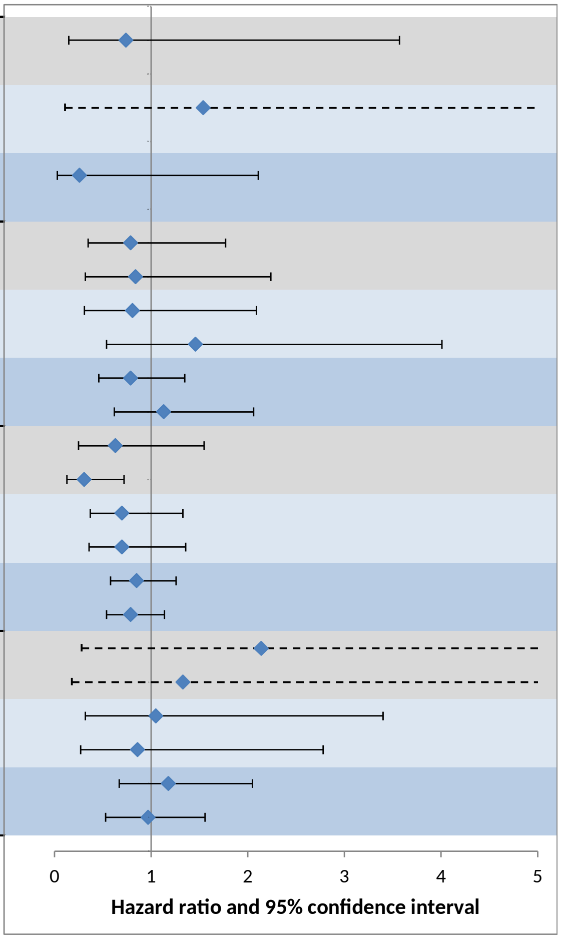

* No. non-breast cancer deaths in referent group, risk <1.0%:

Age at diagnosis 35-44: BCSC-1 N=14; BCRAT N=5; BCSC-5 N=19
Age at diagnosis 45-54: BCSC-1 N=9; BCRAT N=9; BCSC-5 N=29
Age at diagnosis 55-69: BCSC-1 N=5; BCRAT N=10; BCSC-5 N=25
Age at diagnosis 70-89: BCSC-1 N=0; BCRAT N=3; BCSC-5 N=12

Note: Deaths with missing covariate information are not included so counts will not add up to Supplemental Figure 4.
