# Supplementary material for: Relationship of Predicted Risk of Developing Invasive Breast Cancer, as Assessed with Three Models, and Breast Cancer Mortality among Breast Cancer Patients
Source: PLoS One. 2016 Aug 25;11(8):e0160966. doi: 10.1371/journal.pone.0160966 (PMC4999085; doi:10.1371/journal.pone.0160966)
Supplement: Supplementary file 10 [file pone.0160966.s010.docx]

Tumor characteristic	Age at diagnosis	BCSC-1 risk model			BCRAT risk model			BCSC-5 risk model		
		0-<1%	1-1.66%	≥1.67%	0-<1%	1-1.66%	≥1.67%	0-<1%	1-1.66%	≥1.67%
AJCC stage >IIA	35-44	0.34 (0.32-0.37)	0.30 (0.24-0.37)	0.19 (0.05-0.32)	0.32 (0.28-0.37)	0.33 (0.23-0.44)	0.45 (0.23-0.67)	0.33 (0.31-0.36)	0.30 (0.23-0.37)	0.29 (0.08-0.51)
	45-54	0.27 (0.24-0.30)	0.27 (0.25-0.30)	0.25 (0.22-0.28)	0.23 (0.20-0.26)	0.26 (0.22-0.29)	0.22 (0.17-0.26)	0.28 (0.26-0.30)	0.26 (0.24-0.28)	0.23 (0.20-0.26)
	55-69	0.19 (0.11-0.27)	0.24 (0.20-0.29)	0.19 (0.17-0.21)	0.18 (0.13-0.23)	0.18 (0.16-0.20)	0.19 (0.17-0.22)	0.21 (0.18-0.24)	0.24 (0.22-0.26)	0.22 (0.21-0.23)
	70-89	0.08 (0.00-0.24)	0.13 (0.06-0.21)	0.16 (0.14-0.18)	0.14 (0.00-0.28)	0.15 (0.12-0.18)	0.15 (0.13-0.17)	0.24 (0.14-0.33)	0.20 (0.17-0.23)	0.17 (0.16-0.18)
	All ages	0.31 (0.29-0.32)	0.27 (0.25-0.29)	0.19 (0.18-0.21)	0.25 (0.22-0.27)	0.20 (0.18-0.21)	0.18 (0.16-0.19)	0.29 (0.27-0.30)	0.24 (0.23-0.26)	0.20 (0.19-0.21)
Interval cancer or clinically detected	35-44	0.68 (0.65-0.71)	0.62 (0.55-0.69)	0.57 (0.39-0.75)	0.64 (0.59-0.69)	0.58 (0.47-0.69)	0.59 (0.35-0.82)	0.67 (0.65-0.70)	0.59 (0.52-0.67)	0.54 (0.27-0.81)
	45-54	0.52 (0.48-0.56)	0.54 (0.51-0.58)	0.51 (0.47-0.55)	0.50 (0.46-0.54)	0.46 (0.42-0.50)	0.48 (0.42-0.54)	0.51 (0.49-0.54)	0.53 (0.50-0.55)	0.50 (0.46-0.53)
	55-69	0.16 (0.07-0.25)	0.39 (0.33-0.46)	0.33 (0.31-0.36)	0.36 (0.29-0.43)	0.33 (0.31-0.36)	0.37 (0.34-0.40)	0.35 (0.31-0.40)	0.36 (0.34-0.39)	0.41 (0.39-0.43)
	70-89	0.25 (0.00-0.55)	0.24 (0.14-0.35)	0.33 (0.30-0.36)	0.24 (0.03-0.44)	0.34 (0.30-0.38)	0.33 (0.30-0.36)	0.39 (0.28-0.51)	0.37 (0.33-0.41)	0.38 (0.36-0.40)
	All ages	0.60 (0.58-0.62)	0.52 (0.49-0.54)	0.37 (0.36-0.39)	0.52 (0.49-0.54)	0.38 (0.36-0.40)	0.37 (0.35-0.39)	0.55 (0.54-0.57)	0.44 (0.42-0.46)	0.41 (0.39-0.42)
ER negative	35-44	0.27 (0.24-0.29)	0.32 (0.25-0.39)	0.10 (0.00-0.21)	0.24 (0.19-0.28)	0.23 (0.13-0.32)	0.28 (0.07-0.48)	0.28 (0.25-0.30)	0.22 (0.15-0.28)	0.19 (0.00-0.38)
	45-54	0.25 (0.22-0.28)	0.20 (0.18-0.23)	0.18 (0.15-0.21)	0.22 (0.19-0.25)	0.18 (0.15-0.20)	0.17 (0.13-0.21)	0.25 (0.23-0.27)	0.21 (0.19-0.23)	0.20 (0.17-0.22)
	55-69	0.23 (0.14-0.32)	0.23 (0.18-0.28)	0.15 (0.13-0.17)	0.16 (0.11-0.21)	0.17 (0.15-0.19)	0.15 (0.13-0.18)	0.21 (0.18-0.24)	0.21 (0.19-0.23)	0.17 (0.16-0.18)
	70-89	0.11 (0.00-0.32)	0.17 (0.08-0.25)	0.14 (0.11-0.16)	0.33 (0.13-0.54)	0.12 (0.09-0.15)	0.11 (0.10-0.13)	0.23 (0.13-0.33)	0.14 (0.11-0.17)	0.15 (0.13-0.16)
	All ages	0.26 (0.24-0.28)	0.22 (0.20-0.24)	0.15 (0.14-0.16)	0.21 (0.19-0.24)	0.16 (0.15-0.18)	0.14 (0.13-0.15)	0.25 (0.24-0.26)	0.20 (0.19-0.21)	0.16 (0.15-0.17)
